# The herbivore’s dilemma: Trends in and factors associated with heterosexual relationship status and interest in romantic relationships among young adults in Japan—Analysis of national surveys, 1987–2015

**DOI:** 10.1371/journal.pone.0241571

**Published:** 2020-11-09

**Authors:** Cyrus Ghaznavi, Haruka Sakamoto, Shuhei Nomura, Anna Kubota, Daisuke Yoneoka, Kenji Shibuya, Peter Ueda

**Affiliations:** 1 Department of Global Health Policy, Graduate School of Medicine, The University of Tokyo, Tokyo, Japan; 2 Department of Medicine, Washington University School of Medicine, St. Louis, Missouri, United States of America; 3 Department of Tropical Medicine and International Affairs, Tokyo Women’s Medical University, Tokyo, Japan; 4 Division of Biostatistics and Bioinformatics, Graduate School of Public Health, St. Luke’s International University, Tokyo, Japan; 5 Clinical Epidemiology Division, Department of Medicine, Solna, Karolinska Institute, Stockholm, Sweden; Universita degli studi di Padova (Padua University), ITALY

## Abstract

**Background:**

It has been suggested that an increasing proportion of young adults in Japan have lost interest in romantic relationships, a phenomenon termed “herbivorization”. We assessed trends in heterosexual relationship status and self-reported interest in heterosexual romantic relationships in nationally representative data.

**Methods:**

We used data from seven rounds of the National Fertility Survey (1987–2015) and included adults aged 18–39 years (18–34 years in the 1987 survey; sample size 11,683–17,675). Current heterosexual relationship status (married; unmarried but in a relationship; single) was estimated by sex, age group and survey year, with singles further categorized into those reporting interest vs. no interest in heterosexual romantic relationships. Information about same-sex relationships were not available.

**Results:**

Between 1992 and 2015, the age-standardized proportion of 18-39-year-old Japanese adults who were single had increased steadily, from 27.4 to 40.7% among women and from 40.3 to 50.8% among men. This increase was largely driven by decreases in the proportion of married women aged 25–39 years and men aged 30–39 years, while those in a relationship had increased only slightly for women and remained stable for men. By 2015, the proportion of single women was 30.2% in those aged 30–34 years and 24.4% in those aged 35–39 years. The corresponding numbers for men were 39.3% and 32.4%. Around half of the singles (21.4% of all women and 25.1% of all men aged 18–39 years) reported that they had no interest in heterosexual romantic relationships. Single women and men who reported no interest in romantic relationships had lower income and educational levels and were less likely to have regular employment compared to those who reported such an interest.

**Conclusions:**

In this analysis of heterosexual relationships in nationally representative data from Japan, singlehood among young adults had steadily increased over the last three decades. In 2015 around one in four women and one in three men in their thirties were unmarried and not in a heterosexual relationship. Half of the singles reported no interest in romantic relationships and these women and men had lower income and educational levels and were less likely to have regular employment.

## Background

The phenomenon of “herbivore” (soushokukei) young adults in Japan has received considerable attention in the past decade [1–4]. Although its definition is subject to debate [5], the term is broadly used to describe young adults, particularly men, who lack interest in pursuing sex and romantic relationships [6, 7], with such individuals being contrasted against the “carnivores” (nikushokukei) who actively pursue potential partners. It is widely assumed that “herbivorization” is a defining characteristic of young adulthood in Japan [8]. By the same token, marriage rates have been steadily decreasing [9], and in the 2015 National Fertility Survey, 25.9% of never-married women and 30.2% of never-married men (aged 18–34 years) responded that they were not interested in having a romantic relationship with someone of the opposite sex [10].

Despite government efforts to encourage marriage, pregnancy, and childrearing, Japan continues to struggle with low total fertility rates [11], with its population estimated to decrease from 127 million in 2015 to less than 90 million by 2065 [12]. Provided that heterosexual relationships constitute the most common route to family formation, it has been suggested that the herbivorization of young adults contributes to Japan’s low birth rates [6, 13]. Furthermore, individuals in romantic relationships tend to experience better health outcomes and life satisfaction compared to their single counterparts, although these associations depend on the quality of the relationship [14–16]. If a large number of individuals live without intimate relationships, this may also have implications for public health.

Important knowledge gaps regarding the herbivore phenomenon remain. Although a decline in marriage rates has been observed [9], the extent to which this may have translated into singlehood among young adults is uncertain as unmarried individuals may be in romantic relationships; thus, trends in relationship status have not been assessed [10]. In addition, despite substantial media attention and public discourse focusing on herbivore adults [1–4, 6], little is known about those who remain single and uninterested in romantic relationships.

In this study, we used data from seven rounds of the National Fertility Survey, conducted between 1987 and 2015, to assess trends in heterosexual relationship status (married; unmarried but in a relationship; single) among young Japanese adults. Furthermore, we estimated the proportion of the singles that reported no interest in heterosexual romantic relationships and identified socioeconomic factors associated with relationship status and interest in relationships.

## Methods

### Data sources

We used data from the National Fertility Survey of Japan, conducted in 1987, 1992, 1997, 2002, 2005, 2010, and 2015. The survey, which is described in detail elsewhere [10, 17, 18], is carried out by The National Institute of Population and Social Security Research (IPSS), under the Japanese Ministry of Health, Labour and Welfare, to collect nationally representative data on topics related to marriage and childbirth. In brief, each survey used stratified cluster sampling with districts in the Population Census of Japan as primary sampling units and included two national sub-surveys: one for married couples in which the wife was under 50 years of age (with the wife providing information about the husband) and one for unmarried women and men aged 18–49 years (the 1987 survey of unmarried adults included those aged 18–34 years) [10]. Survey administrators provided participants with a self-administered questionnaire during a home visit; the questionnaire was returned upon completion in a sealed envelope during a follow-up visit. Between 1987 and 2015, the response rate ranged between 70.0 and 83.8% (mean 76.1%) among unmarried respondents and 85.7 and 92.5% (mean 88.3%) among married couples [10].

Information on the number of individuals in the Japanese population by age, sex, and marital status was obtained from the Population Census of Japan (1985–2015) [9]. We used these data for calculation of sample weights, as described below. The study was approved by the Regional Ethics Committee at The University of Tokyo, Japan. Informed consent was not required for secondary use of data from the National Fertility Survey. All analyses were performed on anonymized data.

### Study population

We included married and unmarried women and men, aged between 18 and 39 years. We excluded unmarried individuals with unknown relationship status, as defined below. Across the surveys, the proportion of excluded participants ranged from 1.6 to 5.7% (5.0 to 12.0% of the unmarried respondents) among women and from 2.5 to 7.0% (6.0 to 12.7% of the unmarried respondents) among men. The demographic characteristics of unmarried participants with known relationship status included in the analyses were similar to the excluded participants ([S1 and S2 Tables in S1 File]). The final sample size ranged between 11,683 (6,215 unmarried) and 17,675 (8,008 unmarried) across survey years ([S3 Table in S1 File]).

Sample weights were used to adjust for differential probabilities of non-response and unknown relationship status by age, sex, and marital status and calculated based on data from the Population Census of Japan, as described in S1 File [9]. In brief, after exclusion of survey participants with missing data on relationship status, sample weights, standardized to the age-distribution of 2015, were calculated so that each survey was representative of the Japanese population with respect to sex, age and marital status.

### Relationship status and interest in romantic relationships

As the National Fertility survey did not include questions regarding same-sex relationships, and same-sex marriage is not established in Japan, our analyses were limited to heterosexual relationships. Participants’ relationship status was categorized into (1) married, (2) unmarried but in a relationship, and (3) single. All participants in the survey of married individuals were considered as married. The sub-survey of unmarried participants included the question, “Are you currently in a relationship with someone of the opposite sex?” [10]. Those who responded that they were in a relationship with a romantic partner (*koibito*) or had a fiancé were categorized as being in a relationship. Those who answered that they were not in a relationship were categorized as single, as described S1 File. In the 2010 and 2015 surveys, participants who responded that they were not in a relationship were asked whether they had an interest in having a romantic relationship. Based on their response to this question, participants were categorized into those with interest vs. no interest in romantic relationships with someone of the opposite sex (S1 File). The questions were worded consistently in all surveys.

### Statistical methods

All analyses were stratified by sex and performed in Stata version 14.0 (StataCorp LP, College Town, TX) and *R* version 3.3.2. First, we used sample weights, standardized to the age distribution of 2015, to estimate the age-standardized proportions of young adults in each category of relationship status in the full age range (18–39 years) and by age group (18–24, 25–29, 30–34, and 35–39 years) in the seven surveys. Analyses were not performed for the age groups 18–39 years and 35–39 years in 1987 because unmarried individuals older than 34 years were not included in this survey. For each category of relationship status, we assessed trends across the study period by calculating age-adjusted odds ratios (aOR) using logistic regression with survey year and age as independent variables and the relationship status of interest (yes/no) as the dependent variable. In the 2010 and 2015 surveys, we calculated the proportion of single adults with interest and no interest in a relationship. For these analyses, singles with unknown interest in relationships (*n* = 1,032 [weighted proportion of singles 17.6%] in 2010; *n* = 893 [19.2%] in 2015) were excluded, as described in the S1 File.

Next, applying the sample weights, we described the population characteristics in the 2015 survey by relationship status and interest in relationships. We selected, *a priori*, variables that we hypothesized could be associated with relationship status and interest in relationships: education, occupational status, annual income, hours worked/week (for regular employees only), heterosexual experience, wish to get married during the lifetime, region of residence, and population size/density of residence; the definitions and categorizations of these variables are shown in S1 File (S4 Table in S1 File) [6, 18–22]. We then used sample weights that were standardized to the age distribution of married individuals (S1 File) to assess the age-standardized characteristics across relationship status and interest in relationships and calculated the relative risk for the investigated category of the variable for each of the groups vs married individuals. Differences in the distribution of characteristics across the categories were assessed using the Chi-squared test. Participants with missing data for any given variable were few (≤ 3.5%, S1 File [S4 Table in S1 File]) and excluded from the analyses by variable. P-values of < 0.05 and aOR confidence intervals that did not include 1 were considered as statistically significant.

### Post-hoc analyses

We performed two sets of post-hoc analyses, which are described in S1 File. First, as we found that a substantial proportion of singles reporting no interest in romantic relationship indicated that they wished to get married in their lifetime, we assessed self-reported reasons for remaining single in this group. Second, in order to compare the proportion of the Japanese population that is single to that of other high-income nations, we estimated the proportion of single women and men using nationally representative survey data from Britain (Natsal-3) [23] and the US (General Social Survey) [24].

## Results

### Heterosexual relationship status, 1987–2015

**Fig 1:**
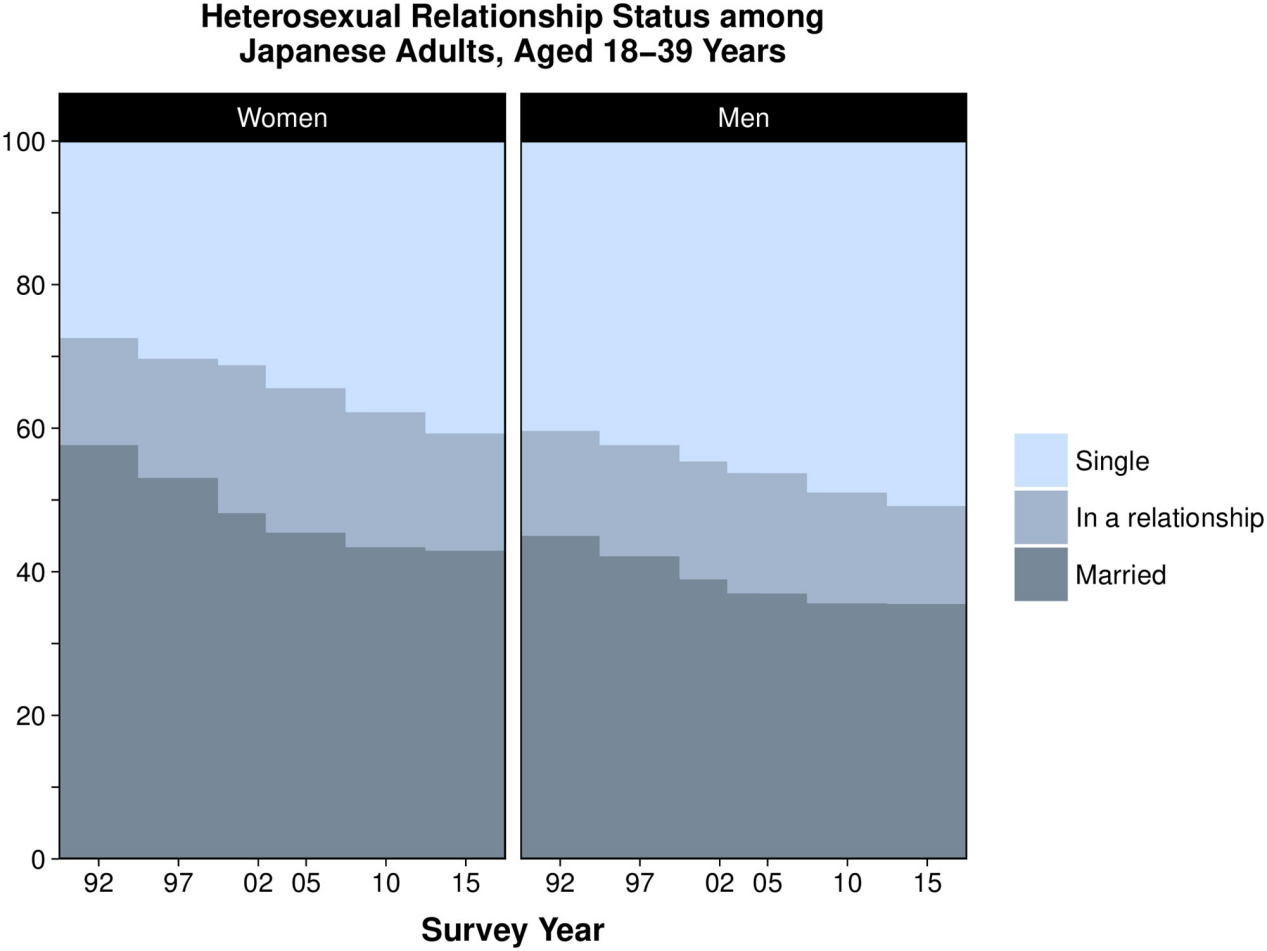
Trends in heterosexual relationships status among Japanese women and men, aged 18–39 years, 1992–2015. Proportions in 1992–2010 were standardized to the age distribution of 2015.

**Fig 2:**
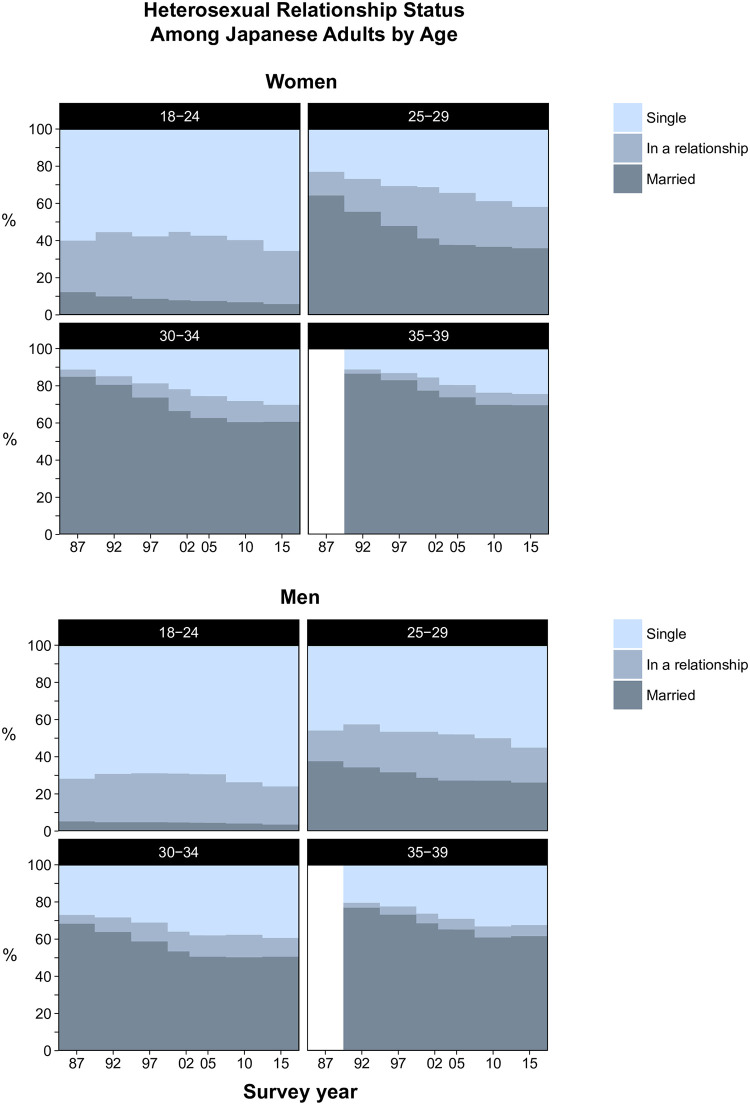
Trends in heterosexual relationships status among Japanese women and men by age group, 1987–2015. Proportions in 1987–2010 were standardized to the age distribution of 2015.

Trends in heterosexual relationship status for the total age range are shown in Fig 1 (raw data in S5 and S6 Tables in S1 File) and by age group in Fig 2 ([S7 Table in S1 File]) for women and Fig 2 ([S8 Table in S1 File]) for men.

The age-standardized proportion of singles increased substantially from 27.4% in 1992 to 40.7% in 2015 for women (aOR per year since 1992, 1.030 [95% CI 1.027–1.033]; average absolute percent increase per 10 years, 5.8%) and from 40.3 to 50.8% for men (aOR 1.022 [1.019–1.025], absolute increase per 10 years, 4.6%), while the proportion who were unmarried but in a relationship increased only slightly for women (14.9% in 1992 vs. 16.3% in 2015, [aOR 1.009 [1.005–1.012], absolute increase per 10 years, 0.6%]) and remained stable among men (14.6 vs. 13.6% [aOR 0.999 [0.995–1.002]]. Contrastingly, there was a large decrease in the proportion of married individuals aged 18–39 years, from 57.7% in 1992 to 42.9% in 2015 for women (aOR 0.960 [0.957–0.963], absolute decrease per 10 years 6.4%) and from 45.0 to 35.5% for men (aOR 0.974 [0.971–0.977], absolute decrease per 10 years 4.1%).

When analyzed by age group, the proportion who were single increased only slightly among both women (60.0% in 1987 vs. 65.6% in 2015; aOR 1.007 [1.003–1.011], absolute increase per 10 years, 2.0%) and men (71.8% vs. 75.9%; aOR 1.008 [1.003–1.012], absolute increase per 10 years, 1.5%) aged 18–24 years. Among women, the proportion single steadily and substantially increased among those aged 25–29 years (23.0% in 1987 vs. 41.9% in 2015; aOR 1.031 [1.026–1.036], absolute increase per 10 years, 6.8%), 30–34 years (11.3% vs. 30.2%; aOR 1.045 [1.039–1.051], absolute increase per 10 years, 6.8%), and 35–39 years (11.2% in 1992 vs. 24.4% in 2015; aOR 1.046 [1.039–1.054], absolute increase per 10 years, 5.7%). Although not as pronounced as in women, there was a large increase in the proportion single also among men aged 25–29 years (45.8% vs. 55.1%; aOR 1.013 [1.009–1.018], absolute increase per 10 years, 3.3%), 30–34 years (26.9% in 1987 vs. 39.3% in 2015; aOR 1.023 [1.018–1.028], absolute increase per 10 years, 4.4%), and 35–39 years (20.4% in 1992 vs. 32.4% in 2015; aOR 1.032 [1.026–1.038], absolute increase per 10 years, 5.2%). These increases in singlehood among women and men were largely driven by decreases in the proportion married, which were only partly compensated for by slight increases in the proportion who were in a relationship.

### Interest in heterosexual romantic relationships, 2010 & 2015

**Fig 3:**
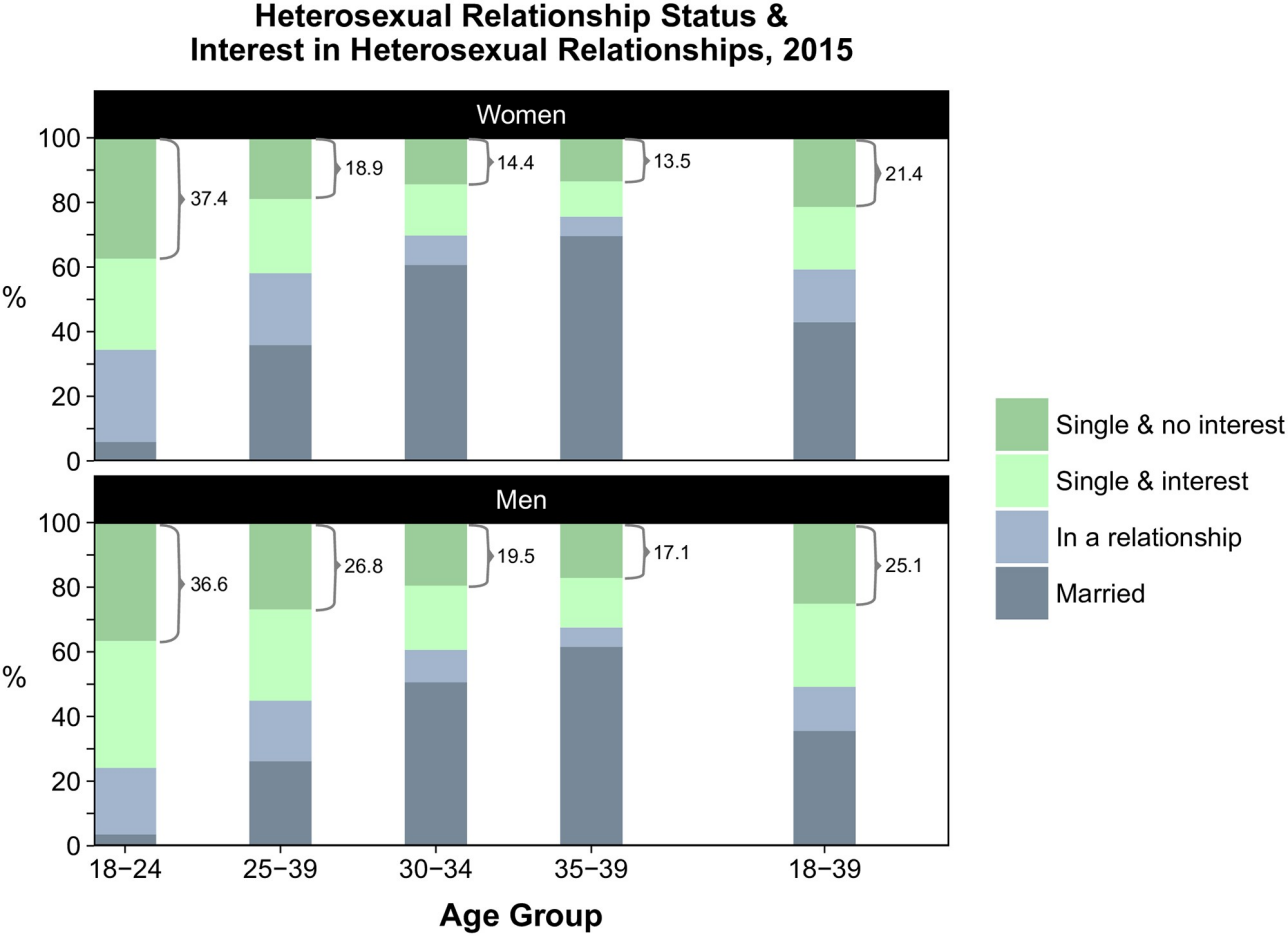
Heterosexual relationship status and interest in heterosexual romantic relationships by age group among women and men in 2015.

The proportion of women and men in each relationship status category as well as reported interest in romantic relationships by age group are shown in Fig 3 (2015) and S1 File (S1 Fig in S1 File [2010] and S7 Table in S1 File [raw data]). Overall, a higher proportion of men than women reported no interest in relationships and, in each of the age groups, the findings were largely similar in 2010 and 2015. In 2015, around half of the singles, or 21.4% of all women and 25.1% of all men aged 18–39 years, reported no interest in romantic relationships. No interest in relationships was more common in younger age groups and decreased with age: 37.4% of women and 36.6% of men aged 18–24 years were single and reported no interest, but this proportion fell to 13.5% and 17.1%, respectively, for those aged 35–39 years.

### Population characteristics by heterosexual relationship status and interest in heterosexual romantic relationships, 2015

The sample-weighted characteristics of the 2015 National Fertility Survey participants by relationship status and interest in relationships are shown in Table 1 (women) and Table 2 (men), and the age-standardized characteristics are shown in S1 File (S8 Table in S1 File [women] and S9 Table in S1 File [men]). Of the single women with no interest in relationships, 36.8% had heterosexual experience and 69.2% wished to get married in their lifetime. The corresponding numbers for single men with no interest in relationships were 44.6% and 65.7%. Married women and men tended to be older than those who were unmarried.

**Table 1:** Weighted population characteristics of women by relationship status and interest in relationship in 2015. Numbers are shown in percent.

	*Married*	*Relationship*	*Single & interested*	*Single & no interest*	*p-value*	*p-value (age-standardized)*
***Age group***					< 0.0001	-
18–24	3.7	48.0	39.0	46.8		
25–29	17.3	28.2	24.8	18.5		
30–34	33.9	13.5	19.3	15.8		
35–39	45.1	10.3	16.9	18.9		
***Education***					< 0.0001	0.0017
High school of less	34.3	29.9	22.5	36.6		
Vocational school or short college	39.3	32.3	35.3	27.7		
Undergraduate studies	24.2	36.9	41.0	33.8		
Graduate studies	2.2	1.0	1.2	1.9		
***Occupational Status***					< 0.0001	< 0.0001
Regular employee	25.9	52.2	51.5	34.2		
Part-time or temporary worker	32.0	23.2	20.3	27.1		
Business owner or family business	5.0	2.1	1.6	2.5		
Unemployed	36.9	5.8	6.6	10.8		
Student	0.2	16.7	20.0	25.4		
***Working Hours (per week)***^a^					< 0.0001	0.0001
0–40	55.3	37.3	37.5	44.1		
41–60	43.7	59.1	56.4	53.4		
> 60	1.0	3.6	6.1	2.5		
***Annual Income (in Japanese Yen 10*,*000s)***					< 0.0001	< 0.0001
0	61.8	36.6	38.4	51.4		
100–299	23.3	41.8	40.8	36.4		
300–499	11.9	19.7	18.6	10.8		
500–799	2.6	1.8	2.3	1.3		
≥ 800	0.4	0.1	0.0	0.0		
***Wish to Get Married in Lifetime***					< 0.0001	< 0.0001
No	-	2.8	1.5	30.8		
Yes	-	97.2	98.5	69.2		
***Heterosexual Experience***					< 0.0001	< 0.0001
Yes	100	87.2	52.5	37.6		
No	0	12.8	47.5	62.4		
***Region of Residence***					0.0009	0.1365
Hokkaido	3.4	3.5	4.0	3.6		
Tohoku	6.7	8.4	5.8	9.2		
Kanto	31.6	30.1	37.4	33.9		
Chubu	21.0	20.5	17.1	17.3		
Kinki	15.1	18.6	16.2	15.7		
Chugoku/Shikoku	11.0	7.2	8.4	8.6		
Kyushu/Okinawa	11.2	11.7	11.1	11.6		
***Area of Residence*: *Population*** *Size* and Density					0.0183	0.2757
Non-densely inhabited area	28.5	26.8	25.3	29.4		
< 200,000	24.5	24.4	20.8	25.9		
200,000 to < 1,000,000	27.7	30.6	31.1	27.1		
≥ 1,000,000	19.3	18.2	22.8	17.6		
***N* unweighted/weighted**	3009/2601	887/984.6	810/923.1	885/1019		

^a^. Among participants with regular employment.

**Table 2:** Weighted population characteristics of men by heterosexual relationship status and interest in romantic relationships in 2015. Numbers are shown in percent.

	*Married*	*Relationship*	*Single & interested*	*Single & no interest*	*p-value*	*p-value (age-standardized)*
***Age group***					< 0.0001	-
18–24	2.8	41.9	42.0	40.0		
25–29	15.2	28.3	22.9	22.3		
30–34	33.9	17.7	18.1	18.2		
35–39	48.1	12.2	17.1	19.4		
***Education***					< 0.0001	0.0052
High school of less	41.5	34.4	35.8	43.1		
Vocational school or short college	18.8	15.6	16.8	21.0		
Undergraduate studies	33.7	43.0	41.5	32.5		
Graduate studies	6.0	7.1	5.9	3.4		
***Occupational Status***					< 0.0001	< 0.0001
Regular employee	85.8	63.2	53.2	42.6		
Part-time or temporary worker	4.1	11.8	14.3	17.6		
Business owner or family business	9.2	5.3	4.9	4.3		
Unemployed	0.7	2.7	5.4	14.4		
Student	0.2	17.0	22.1	21.1		
***Working Hours (per week)***^a^					< 0.0001	0.1091
0–40	21.4	26.6	26.5	32.2		
41–60	64.0	65.5	63.6	59.8		
> 60	14.5	7.9	9.9	8.0		
***Annual Income (in Japanese Yen 10*,*000s)***					< 0.0001	< 0.0001
0	6.8	33.0	40.7	48.2		
100–299	16.6	26.0	24.8	28.2		
300–499	44.5	32.7	27.4	19.7		
500–799	27.2	7.7	6.7	3.8		
≥ 800	5.0	0.7	0.4	0.1		
***Wish to Get Married in Lifetime***					< 0.0001	< 0.0001
No	-	1.4	2.6	34.3		
Yes	-	98.6	97.4	65.7		
***Heterosexual Experience***					< 0.0001	< 0.0001
Yes	100	93.5	57.2	44.9		
No	0	6.5	42.8	55.1		
***Region of Residence***					0.0003	0.0059
Hokkaido	3.9	2.6	2.4	3.7		
Tohoku	6.6	6.3	6.1	6.5		
Kanto	30.7	36.3	39.1	38.8		
Chubu	20.5	18.5	19.0	16.9		
Kinki	14.8	16.8	14.7	15.7		
Chugoku/Shikoku	11.5	11.1	10.1	8.8		
Kyushu/Okinawa	11.9	8.6	8.6	9.6		
***Area of Residence*: *Population Size* and Density**					0.4136	0.8177
Non-densely inhabited area	28.8	29.6	30.3	27.9		
< 200,000	25.1	21.7	22.5	24.6		
200,000 to < 1,000,000	27.7	27.1	27.9	29.6		
≥ 1,000,000	18.4	21.5	19.4	17.9		
***N* unweighted/weighted**	2459/2000	671/767.2	1046/1199	1023/1169		

^a^. Among participants with regular employment.

Married women (36.9%), as well as those who were single and reported no interest in relationships (10.8%), were more likely to be unemployed than women in relationships (5.8%) and single women with interest in relationships (6.6%). Moreover, single women who reported no interest in relationships were more likely to have an education of high school or less (36.6%) when compared to single women with interest in relationships (22.5%). Among regular employees, 55.3% of married women worked 40 hours or less per week while this proportion was 37.3%, 37.5% and 44.1% among those in a relationship, single with interest and single without interest, respectively. The variations across the groups were statistically significant in analyses before and after age-standardization.

Among men, occupational status and income varied substantially across relationship status groups. The proportion with regular employment was highest among married respondents (85.8%) and decreased stepwise for those in a relationship (63.2%), single with interest in relationships (53.2%), and single with no interest (42.6%). A similar pattern was observed for annual income, with married men having the highest income and singles with no interest in relationships having the lowest income. For example, while 32.2% of married men had an annual income of ≥5 million Japanese Yen (JPY), this proportion was 8.4%, 7.1% and 3.9% among those in a relationship, single with interest and single without interest, respectively. Moreover, single men with no interest in relationships had lower educational levels compared to those in the other relationship categories. These patterns persisted after age-standardization.

### Post-hoc analyses

Of the singles with no interest in relationships, the two most common reasons for not getting married were “do not feel the need to marry yet” (women: 37.1%, men: 33.7%) and “have not met a suitable partner” (women: 39.4%, men: 33.2%). ([S2 Fig in S1 File]).

The estimated proportion of the population that is single in Britain and the US is shown in S1 File (S10 Table in S1 File).

## Discussion

Intimate relationships constitute an important component of human fertility and life satisfaction and are associated with better health outcomes [6, 13–16]. As such, a purportedly decreasing interest in sex and romantic relationships has brought young Japanese adults under national scrutiny and fostered unease in light of the country’s rapidly ageing population, low birth rates, and large number of individuals living in social isolation [25, 26]. In this study, which is the first to investigate the phenomenon of so-called “herbivore” young adults in nationally representative data in Japan, we assessed changes in, and sociodemographic factors associated with, heterosexual relationship status and reporting of no interest in heterosexual romantic relationships.

Between 1992 and 2015, the age-standardized proportion of 18-39-year-old Japanese adults who were single (unmarried and not in a heterosexual relationship) had increased steadily, from 27.4 to 40.7% among women and from 40.4 to 50.8% among men, with these numbers corresponding an increase of 2.2 million single women and 1.7 million single men in the investigated age range. The increase was largely driven by increases in singlehood among women aged 25–39 years and men aged 30–39 years. By 2015, around one in four women and one in three men in their thirties were single. Our findings show that the decline in marriage among young adults [9] indeed reflects an increase in singlehood as the proportion who were unmarried and in a relationship rose only slightly among women and remained stable among men. Notably, being in a relationship became increasingly uncommon with higher age and among adults in their thirties, this group was so small that the proportion who were unmarried largely equaled the proportion who were single.

Around one in five women and men aged 18–39 years in 2015 were single while reporting that they had no interest in heterosexual romantic relationships. It should be assumed that a proportion of these individuals have interest, or are currently in, non-heterosexual sexual or romantic relationships. Moreover, the term “*kousai”* which was used in the question about interest in romantic relationships was not defined in the National Fertility Survey. This term is commonly used to refer to the act of engaging in a relationship with a romantic/sexual partner, but as with the word “dating,” the level of commitment to such a relationship is uncertain; it is possible that a proportion of those who answered that they had no interest in a romantic relationship may have had interest in sexual or romantic pursuits but preferred not to be in a stable relationship at the time of survey participation. However, over half of women and men who reported no interest in heterosexual romantic relationships had no heterosexual experience, indicating that many of these individuals are indeed sexually inactive.

While interpretation of the results on income and employment status among women is made difficult by the relatively large proportion of married women who are housewives [27], we note that single women without interest in romantic relationship had lower educational levels and income than single women with such an interest. For men, there was a pronounced gradient in income and occupational status across categories of relationship status and interest in relationships. For example, the proportion with regular employment was highest among married men (85.8%) and decreased stepwise for those in a relationship (63.2%), single with interest in relationships (53.2%), and single with no interest (42.6%). In age-adjusted analyses, married men were twice as likely to have an annual income of JPY 5 million as compared to those in a relationship and those who were single with an interest in relationship and more than three times as likely to have such an income compared to those without interest in romantic relationships.

The herbivore stereotype has typically been portrayed as a timid individual (most of the time male), seemingly indifferent to sex and romance; in other words, “herbivore” is largely described as a type of personality or a set of behaviors secondary to sexual interests [7, 8, 28]. This notion is challenged by the association of lower education, low income, and a lack of regular employment with reporting no interest in relationships among single women and men. It is likely that aspirations and outcomes in the labor and partner market have common determinants such as personality, values, physical appearance and physical and mental health status, and the associations may not represent causality. Nonetheless, high and stable incomes are important predictors of appeal in the partner market, particularly for men [10, 29–31], indicating that individuals with lower income may have difficulties in finding romantic partners. In addition, one could speculate that precarious employment and financial insecurity may contribute to decreased resources, motivation, and opportunities for pursuing romantic relationships [18]. Indeed as shown in our study and in previous analyses [32], income is strongly associated with marriage among Japanese men. Notably, in a study using longitudinal data (2002–2006) from a nationally representative sample of women [33], those with higher earnings (before marriage) were more likely to marry at any age than those with lower earnings. Moreover, women with a university or junior college degree were more likely to be married at the age of 38 years as compared with those with a high school degree or less, indicating that, perhaps contrary to popular belief [34], high earning potential and education constitute advantages in the partner market also for Japanese women. As such, it could be hypothesized that for some women and men, the reporting of no interest in romantic relationships may represent an adjustment of their expectations, or resignation, given their current lack of access to romantic partners and inability to pursue them, while they would be otherwise interested if they were to meet suitable partners. In support of this hypothesis, we found that around two thirds of the women and men reporting no interest in romantic relationships answered that they wished to get married in their lifetime and that around one third reported that a major reason for not getting married was that they had not met a suitable partner. It should also be noted that the proportion who reported no interest in romantic relationships in 2015 decreased with age and the proportions were largely similar in 2010 vs. 2015, indicating that some individuals get married regardless of their reported interest in romantic relationships.

Although there are other types of social connections which may provide benefits to health and life satisfaction [35–39], and while some individuals may prefer to live without romantic relationships, the large proportion of young adults that has lost interest, given up, or find it hard to form romantic relationships may have important implications for public health and fertility. Our findings indicate a possibility that policy measures aimed at improving employment opportunities and addressing economic disadvantage may also lead to increased interest in romantic relationships and marriage. Moreover, income and employment status are considered as important spouse-selection criteria in Japan, especially for women who evaluate potential husbands [10]. Given that most unmarried individuals wish to get married and the most common reason for staying single is that no suitable partner is available [10], it could be speculated that some individuals are reluctant to form romantic relationships with partners who do not fulfill their partner-selection criteria for marriage (e.g. with respect to income and employment status) but who are otherwise of romantic and sexual interest. This hypothesis is supported by our finding that almost no individuals in their late thirties were unmarried and in a romantic relationship, implying that the promotion of marriage as the most socially acceptable form of relationship between adults and a necessary step for co-habitation and family formation could constitute a barrier to forming romantic relationships in Japan.

While the decline in marriage rates has been established in many high-income countries [40], data on relationship status among unmarried individuals is scarce. A situation similar to that in Japan may be ongoing in South Korea, where a national survey found that 68% of unmarried women and 74% of unmarried men aged 20–44 years were not in heterosexual relationships and that young adults listed lack of money as an important reason for giving up dating [41, 42]. In the US and Europe, higher earnings and education have been associated with a higher likelihood of marriage among both women and men [43–45], although it is not known how these factors influence interest in romantic relationships. We found that the proportion of Japanese women who were single was 65.6% (18–24 years), 41.9% (25–29 years), 30.2% (30–34 years), and 24.4% (35–39 years), and the proportion of men who were single was 75.9% (18–24 years), 55.1% (25–29 years), 39.3% (30–34 years), and 32.4% (35–39 years). Our analyses of nationally representative survey data from Britain (Natsal-3, 2010–2012) and the US (General Social Survey, 2012–2018) showed that singlehood tends to be less common in these countries. In Britain, the proportion of women who were single (not married/living together with partner and not in a stable relationship) was 41.5% (18–24 years), 23.6% (25–29 years), 16.3% (30–34 years), and 14.0% (35–39 years). The corresponding numbers for men were 52.6% (18–24 years), 32.5% (25–29 years), 14.7% (30–34 years), and 11.8% (35–39 years). In the US, the proportion of women who were single was 62.6% (18–24 years), 25.2% (25–29 years), 20.0% (30–34 years), and 16.6% (35–39 years). The corresponding numbers for men tended to be closer to those we observed in Japan: 81.4% (18–24 years), 55.8% (25–29 years), 35.9% (30–34 years), and 22.0% (35–39 years). Of note, however, are the slightly different definitions of single used (S1 File), and that the estimates for Britain and the US accounted for any stable relationship, regardless of the gender of the partner, while only heterosexual relationships were considered in Japan.

Our study has limitations. First, as data on relationship status and interest in romantic relationships was self-reported, findings may have been affected by social desirability bias [46]; the risk of such a bias, however, may have been mitigated by the survey’s use of self-administered questionnaires [47]. Second, although the response rate in the National Fertility Survey was high (70.0–83.8% among unmarried individuals and 85.7–92.5% among married couples) and the sample was weighted so that it was representative of the Japanese population in terms of sex, age, and marital status, non-response might have introduced bias in our results. Third, the large sample size may have resulted in small differences in the investigated variables being statistically significant and interpretation of the findings should consider the absolute differences. Fourth, because the data were cross-sectional, we could not assess temporality of the associations of sociodemographic variables with relationship status and interest in romantic relationships. Finally, as the questions asked in the National Fertility Survey were limited to heterosexual relationships, we could not assess other types of relationships. Data on gay, lesbian, bisexual, and transgender groups in Japan is scarce [48], and questions targeted to the experiences of these groups should be included in future surveys.

## Conclusions

The proportion of young Japanese adults who are single (unmarried and not in a heterosexual relationship) has increased steadily during the past three decades; in 2015, one in four women and one in three men in their thirties were single. Around half of the singles, or one in five women and one in four men, aged 18–39 years, reported that they had no interest in romantic relationships with someone of the opposite sex. Among single women and men, those who reported no interest in heterosexual romantic relationships had lower income and educational levels and were more likely have no regular employment. The large proportion of young adults who have lost interest, given up, or find it hard to form romantic relationships may have important implications for public health and fertility in Japan.

## Supporting information

S1 File Additional methods and data. (DOCX)
