# Supplementary material for: The herbivore’s dilemma: Trends in and factors associated with heterosexual relationship status and interest in romantic relationships among young adults in Japan—Analysis of national surveys, 1987–2015
Source: PLoS One. 2020 Nov 9;15(11):e0241571. doi: 10.1371/journal.pone.0241571 (PMC7652314; doi:10.1371/journal.pone.0241571)
Supplement: S1 File — (DOCX) [file pone.0241571.s001.docx]

**S1 File: Supplementary Material**

Ghaznavi C, Sakamoto H, Nomura S, Kubota A, Yoneoka D, Shibuya K, Ueda P*.
The Herbivore’s Dilemma: Trends in and Factors Associated with Heterosexual Relationship Status and Interest in Romantic Relationships Among Young Adults in Japan - Analysis of National Surveys, 1987-2015.*

**Definition of relationship status and interest in romantic relationships among unmarried survey participants**

In each round of the National Fertility Survey, 1987-2015, unmarried survey participants were asked the question:

*"Are you currently in a relationship with someone of the opposite sex?”*

Possible answers:

- *I have no relationship with someone of the opposite sex*.
- *I have a friendship with someone of the opposite sex.*
- *I have a relationship with a romantic partner of the opposite sex*
- *I have a fiancée.*

Participants who answered that they had a relationship with a romantic partner or a fiancée were considered as being in a relationship. Those who answered that they had a friend or had no relationship with someone of the opposite sex were considered as single. Participants who answered that they had a friend of the opposite sex were considered as being single because the context and the phrasing of the question and answer were deemed to not imply a sexual/romantic relationship.

In the 2010 and 2015 surveys, those who answered that they had no relationship with someone of the opposite sex were asked:

*“Interest in a relationship?”*

Possible answers:

- *Have an interest in having a relationship*
- *Don’t have an interest in having a relationship with someone of the opposite sex*

The participants who had answered this question were categorized accordingly.

Because the participants who had answered that they had a friend of the opposite sex (categorized as single as described above) were not asked whether they had an interest in having a relationship, they could not be categorized with respect to interest in relationships.

**Calculation of the proportion with interest in romantic relationships**

In each survey, we estimated the proportion of the women and men, respectively, who were married, in a relationship and single in the total age range (18-39 y) and by age group. In the surveys of 2010 and 2015, we further estimated the proportion of those who were single who reported that they had vs. had no interest in relationships. The status of interest in relationship could not be assessed in some of the single women and men because they had not answered this question, or because they were not asked the question as they had responded that they had a friend of the opposite sex, as shown in the table below.

|  | *Women* | | | | *Men* | | | |
| --- | --- | --- | --- | --- | --- | --- | --- | --- |
|  | *Total n single* | *Single and missing information on interest in romantic relationships, n (% [weighted %])* | | | *Total n single* | *Single and missing information on interest in romantic relationships, n (% [weighted %])* | | |
| Survey  year |  | Not answered question | Friend of the opposite sex | Total |  | Not answered question | Friend of the opposite sex | Total |
| 2010 | 2643 | 53 (2.0 [2.0]) | 479 (18.1 [18.0]) | 532 (20.1 [20.0]) | 3185 | 56 (1.8 [1.8]) | 444 (13.9 [13.9]) | 500 (15.7 [15.7]) |
| 2015 | 2155 | 212 (9.8 [9.9]) | 248 (11.5 [11.4]) | 460 (21.3 [21.2]) | 2502 | 238 (9.5 [9.5]) | 195 (7.8 [7.8]) | 433 (17.3 [17.4]) |

We estimated the proportion of the singles who had interest and no interest in a relationship, respectively, after exclusion of the single participants with no information regarding this variable. In order to estimate the proportion of the total population (including also those who were married and those who were in a relationship) who were single and had no interest in relationships, we then multiplied the proportion of the total population that was single with the estimated proportion of singles who had no interest in relationships. Calculation of 95% CI was therefore not performed for these proportions.

**Calculation of sample weights**

The methods for calculation of sample weights has described in detail elsewhere [1]. Sample weights, which were defined as the inverse of the probability of being sampled according to age (1-year increments) and marital status in the Population Census of Japan, were calculated separately for men and women by using population weights and survey weights. Due to low numbers, married individuals between the ages 18 and 22 years were considered as one group. The sample weights were standardized to the age-distribution of 2015, as this was the year with the most recent data.

For each sex, the population weight for any one age and marital status group was calculated as the proportion of the total number of adults (of the same sex), aged 18-39 years, in the census who belonged to that group. Population weights were standardized to the age distribution of 18-39 year-old adults, by sex, in the 2015 Population Census as follows:

*Population weight = Proportion of the age group within the marital status category (for a given year) * proportion of total n of adults aged 18-39 years within the age group in 2015.*

Survey weights were calculated as the proportion of the total number of 18-39-year-old survey participants, by sex, within the age and marital status group, after exclusion of participants with unknown relationship status.

The age-standardized sample weights for each combination of age and marital status category were calculated for each sex and survey by dividing the population weight by the survey weight. These weights were used to estimate the age-standardized proportion of individuals by relationship status in 1987-2015.

In order to assess age-adjusted differences in population characteristics by relationship status and interest in relationships in 2015, we calculated a separate set of sample weights. We first excluded single participants with unknown interest in relationships as described above, and then calculated sample weights, standardized to the age-distribution of married individuals, for each category of unmarried individuals (in a relationship, single and interested in a relationship, single and not interested in a relationship).

**Analyses of reasons for staying single**

Unmarried participants who had responded that they wished to get married in their lifetime were asked to select up to three reasons for staying single from a list of pre-specified reasons as shown in the table below.

|  | **Reason for staying single** |
| --- | --- |
| A | Still too young for marriage |
| B | Do not feel the need to marry yet |
| C | Currently, prefer to focus on work/school |
| D | Currently, want to spend time on hobbies and pursuing pleasures |
| E | Do not want to lose the freedom and carefree nature of singlehood |
| F | Still have not met a suitable person |
| G | Not good at interacting/going on dates with persons of the opposite sex |
| H | Do not have enough money for marriage |
| I | Do not have prospects for housing for post-marriage life |
| J | Parents and others around me (probably) do not approve of marriage |
| K | Other |
| L | My marriage is already planned |

As a substantial proportion of those who were single and had answered that they had no interest in heterosexual romantic relationships had responded that they wished to get married in their lifetime, we performed a post-hoc analysis in which we assessed reasons for staying single in this group.

Figure S2 shows the weighted proportion of single women (n=885) and men (n=1023) with no interest in romantic relationships who had listed each reason as a top-three reason for not getting married. As participants could list up to three reasons, the sum of the proportions exceeds 100%. The denominator also includes those who did not respond to this question as they had responded that they do not wish to get married in their lifetime.

**Estimation of the proportion single in Britain and the US**

To compare the proportion of the population who were single (not married or in a relationship) in Japan to that of Britain and the US, we performed analyses using data from nationally representative surveys: Natsal-3 (Britain, 2010-2012) and the General Social Survey (US, 2012-2018). These surveys are described in detail elsewhere [2, 3]. Analyses accounted for weighting, clustering and stratification of the data and were performed for each sex separately, for the total age range (18-39 years) and by age group (18-24; 25-29; 30-34; 35-39 y).

In Natsal-3, we included all participants aged 18-39 years (n=7,938) and excluded those with unknown relationship status (n=97); n=7,841 participants were available for the analyses. Single was defined as those not married, not in a registered same-sex civil partnership, not living with partner, and not in a “steady” and ongoing relationship. As such, the participants categorized as single included those who were widowed/divorced/separated/previously lived with partner/never married while not being in a “steady” and ongoing relationship.

In the General Social Survey, we used data from the rounds of 2012, 2014, 2016 and 2018, and included all participants aged 18-39 years (n=3,432); we excluded those with unknown relationship status (n=1,837, of which 1,589 had not been asked the question); n=1,595 participants were available for the analyses. Single was defined as having “no steady partner”. As such, those who were “married with partner”, “living as married” or “partner, not living together” were categorized as being not single.

The results of these analyses are shown in STable 10.

**STable 1.** Number and characteristics of unmarried women in the National Fertility Survey, 1987-2015, who were excluded due to unknown heterosexual relationship status vs. those who were included in the analyses. Numbers are shown in n (%).

| *Survey year* | *1987* | | *1992* | | *1997* | | *2002* | | *2005* | | *2010* | | *2015* | |
| --- | --- | --- | --- | --- | --- | --- | --- | --- | --- | --- | --- | --- | --- | --- |
|  | *Included* | *Excluded* | *Included* | *Excluded* | *Included* | *Excluded* | *Included* | *Excluded* | *Included* | *Excluded* | *Included* | *Excluded* | *Included* | *Excluded* |
| *n (%)* | 2560 (95) | 135 (5) | 3706 (93) | 290 (7) | 3722 (93) | 292 (7) | 3601 (88) | 489 (12) | 3429 (92) | 280 (8) | 3983 (94) | 261 (6) | 3042 (93) | 232 (7) |
| *Age group* |  |  |  |  |  |  |  |  |  |  |  |  |  |  |
| 18-24 | 1914 (75) | 89 (66) | 2520 (68) | 179 (62) | 2227 (60) | 157 (54) | 1823 (51) | 226 (46) | 1684 (49) | 114 (41) | 1858 (47) | 99 (38) | 1377 (45) | 108 (47) |
| 35-29 | 468 (18) | 26 (19) | 722 (19) | 66 (23) | 884 (24) | 78 (27) | 946 (26) | 122 (25) | 835 (24) | 58 (21) | 905 (23) | 57 (22) | 765 (25) | 40 (17) |
| 30-34 | 178 (7) | 20 (15) | 276 (7) | 24 (8) | 391 (11) | 33 (11) | 542 (15) | 78 (16) | 576 (17) | 55 (20) | 670 (17) | 57 (22) | 480 (16) | 34 (15) |
| 35-39 | - | - | 188 (5) | 21 (7) | 220 (6) | 24 (8) | 290 (8) | 63 (13) | 334 (10) | 53 (19) | 550 (14) | 48 (18) | 420 (14) | 50 (22) |
| *Education* |  |  |  |  |  |  |  |  |  |  |  |  |  |  |
| High school of less | 1196 (47) | 72 (53) | 1669 (45) | 156 (54) | 1375 (37) | 121 (41) | 1309 (36) | 209 (43) | 1132 (33) | 98 (35) | 1308 (33) | 93 (36) | 899 (30) | 86 (37) |
| Vocational school or short college | 972 (38) | 35 (26) | 1370 (37) | 84 (29) | 1490 (40) | 117 (40) | 1409 (39) | 165 (34) | 1251 (36) | 92 (33) | 1365 (34) | 83 (32) | 948 (31) | 68 (29) |
| Undergraduate studies | 372 (15) | 25 (19) | 613 (17) | 38 (13) | 826 (22) | 45 (15) | 798 (22) | 84 (17) | 968 (28) | 63 (23) | 1204 (30) | 67 (26) | 1119 (37) | 61 (26) |
| Graduate studies | - | - | - | - | - | - | 38 (1) | 5 (1) | 41 (1) | 4 (1) | 61 (2) | 2 (1) | 41 (1) | 3 (1) |
| Missing | 20 (1) | 3 (2) | 54 (1) | 12 (4) | 31 (1) | 9 (3) | 47 (1) | 26 (5) | 37 (1) | 23 (8) | 45 (1) | 16 (6) | 35 (1) | 14 (6) |
| *Occupational status* |  |  |  |  |  |  |  |  |  |  |  |  |  |  |
| Regular employee | 1687 (66) | 82 (61) | 2473 (67) | 189 (65) | 2016 (54) | 157 (54) | 1604 (45) | 189 (39) | 1375 (40) | 104 (37) | 1629 (41) | 88 (34) | 1367 (45) | 76 (33) |
| Part-time or temporary worker | 106 (4) | 5 (4) | 183 (5) | 11 (4) | 565 (15) | 43 (15) | 810 (22) | 104 (21) | 905 (26) | 72 (26) | 1023 (26) | 55 (21) | 712 (23) | 56 (24) |
| Business owner or family business | 63 (2) | 5 (4) | 54 (1) | 2 (1) | 108 (3) | 12 (4) | 116 (3) | 12 (2) | 77 (2) | 7 (3) | 93 (2) | 8 (3) | 59 (2) | 3 (1) |
| Unemployed | 180 (7) | 15 (11) | 206 (6) | 19 (7) | 235 (6) | 13 (4) | 318 (9) | 32 (7) | 259 (8) | 17 (6) | 350 (9) | 24 (9) | 224 (7) | 17 (7) |
| Student | 496 (19) | 22 (16) | 747 (20) | 51 (18) | 694 (19) | 47 (16) | 603 (17) | 59 (12) | 740 (22) | 31 (11) | 749 (19) | 43 (16) | 572 (19) | 39 (17) |
| Missing | 28 (1) | 6 (4) | 43 (1) | 18 (6) | 104 (3) | 20 (7) | 150 (4) | 93 (19) | 73 (2) | 49 (18) | 139 (3) | 43 (16) | 108 (4) | 41 (18) |
| *Region of residence* |  |  |  |  |  |  |  |  |  |  |  |  |  |  |
| Hokkaido | 108 (4) | 9 (7) | 113 (3) | 7 (2) | 146 (4) | 20 (7) | 144 (4) | 15 (3) | 132 (4) | 12 (4) | 124 (3) | 9 (3) | 106 (3) | 7 (3) |
| Tohoku | 146 (6) | 7 (5) | 244 (7) | 19 (7) | 173 (5) | 21 (7) | 284 (8) | 28 (6) | 241 (7) | 18 (6) | 289 (7) | 17 (7) | 240 (8) | 16 (7) |
| Kanto | 843 (33) | 41 (30) | 1288 (35) | 92 (32) | 1278 (34) | 94 (32) | 1156 (32) | 167 (34) | 1160 (34) | 89 (32) | 1355 (34) | 87 (33) | 1057 (35) | 72 (31) |
| Chubu | 510 (20) | 18 (13) | 711 (19) | 55 (19) | 796 (21) | 62 (21) | 662 (18) | 87 (18) | 605 (18) | 43 (15) | 776 (19) | 48 (18) | 559 (18) | 43 (19) |
| Kinki | 419 (16) | 21 (16) | 631 (17) | 55 (19) | 566 (15) | 41 (14) | 623 (17) | 94 (19) | 587 (17) | 52 (19) | 643 (16) | 46 (18) | 503 (17) | 46 (20) |
| Chugoku/Shikoku | 204 (8) | 7 (5) | 304 (8) | 22 (8) | 381 (10) | 25 (9) | 299 (8) | 39 (8) | 241 (7) | 19 (7) | 312 (8) | 25 (10) | 234 (8) | 24 (10) |
| Kyushu/Okinawa | 330 (13) | 32 (24) | 415 (11) | 40 (14) | 382 (10) | 29 (10) | 433 (12) | 59 (12) | 463 (14) | 47 (17) | 484 (12) | 29 (11) | 343 (11) | 24 (10) |
| *Area of Residence: Population Size and Density* |  |  |  |  |  |  |  |  |  |  |  |  |  |  |
| Non-densely inhabited area | 839 (33) | 37 (27) | 1191 (32) | 108 (37) | 1007 (27) | 85 (29) | 1149 (32) | 149 (30) | 1242 (36) | 98 (35) | 1309 (33) | 105 (40) | 837 (28) | 62 (27) |
| < 200,000 | 689 (27) | 31 (23) | 966 (26) | 64 (22) | 1003 (27) | 93 (32) | 893 (25) | 139 (28) | 814 (24) | 62 (22) | 831 (21) | 59 (23) | 729 (24) | 57 (25) |
| 200,000 to < 1,000,000 | 540 (21) | 42 (31) | 764 (21) | 65 (22) | 917 (25) | 60 (21) | 775 (22) | 98 (20) | 687 (20) | 67 (24) | 1005 (25) | 53 (20) | 888 (29) | 67 (29) |
| ≥ 1,000,000 | 492 (19) | 25 (19) | 785 (21) | 53 (18) | 795 (21) | 54 (18) | 784 (22) | 103 (21) | 686 (20) | 53 (19) | 838 (21) | 44 (17) | 588 (19) | 46 (20) |

When accounting also for married women, the proportion excluded due to missing data on relationship status was 2% (135/8490) in 1987, 3% (290/9141) in 1992, 4% (292/8000) in 1997, 6% (489/8130) in 2002, 4% (280/7328) in 2005, 3% (261/8091) in 2010 and 4% (232/6051) in 2015.

**STable 2.** Number and characteristics of unmarried men in the National Fertility Survey, 1987-2015, who were excluded due to unknown heterosexual relationship status vs. those who were included in the analyses. Numbers are shown in n (%).

| *Survey year* | *1987* | | *1992* | | *1997* | | *2002* | | *2005* | | *2010* | | *2015* | |
| --- | --- | --- | --- | --- | --- | --- | --- | --- | --- | --- | --- | --- | --- | --- |
|  | *Included* | *Excluded* | *Included* | *Excluded* | *Included* | *Excluded* | *Included* | *Excluded* | *Included* | *Excluded* | *Included* | *Excluded* | *Included* | *Excluded* |
| *n (%)* | 3175 (94) | 204 (6) | 4302 (92) | 397 (8) | 4014 (91) | 395 (9) | 3922 (87) | 570 (13) | 3520 (91) | 341 (9) | 4212 (93) | 299 (7) | 3173 (91) | 300 (9) |
| *Age group* |  |  |  |  |  |  |  |  |  |  |  |  |  |  |
| 18-24 | 1966 (62) | 122 (60) | 2509 (58) | 226 (57) | 2118 (53) | 205 (52) | 1932 (49) | 234 (41) | 1398 (40) | 136 (40) | 1728 (41) | 110 (37) | 1301 (41) | 148 (49) |
| 35-29 | 815 (26) | 50 (25) | 983 (23) | 76 (19) | 1083 (27) | 101 (26) | 1032 (26) | 158 (28) | 991 (28) | 82 (24) | 1066 (25) | 56 (19) | 777 (24) | 53 (18) |
| 30-34 | 394 (12) | 32 (16) | 483 (11) | 51 (13) | 504 (13) | 62 (16) | 627 (16) | 116 (20) | 671 (19) | 71 (21) | 792 (19) | 61 (20) | 588 (19) | 46 (15) |
| 35-39 | - | - | 327 (8) | 44 (11) | 309 (8) | 27 (7) | 331 (8) | 62 (11) | 460 (13) | 52 (15) | 626 (15) | 72 (24) | 507 (16) | 53 (18) |
| *Education* |  |  |  |  |  |  |  |  |  |  |  |  |  |  |
| High school of less | 1634 (51) | 121 (59) | 1881 (44) | 229 (58) | 1812 (45) | 193 (49) | 1658 (42) | 301 (53) | 1448 (41) | 180 (53) | 1760 (42) | 145 (48) | 1205 (38) | 134 (45) |
| Vocational school or short college | 388 (12) | 22 (11) | 602 (14) | 44 (11) | 658 (16) | 83 (21) | 791 (20) | 102 (18) | 584 (17) | 44 (13) | 691 (16) | 51 (17) | 573 (18) | 45 (15) |
| Undergraduate studies | 1108 (35) | 54 (26) | 1761 (41) | 101 (25) | 1502 (37) | 105 (27) | 1280 (33) | 131 (23) | 1257 (36) | 84 (25) | 1480 (35) | 77 (26) | 1187 (37) | 92 (31) |
| Graduate studies | - | - | - | - | - | - | 120 (3) | 9 (2) | 181 (5) | 4 (1) | 231 (5) | 6 (2) | 160 (5) | 7 (2) |
| Missing | 45 (1) | 7 (3) | 58 (1) | 23 (6) | 42 (1) | 14 (4) | 73 (2) | 27 (5) | 50 (1) | 29 (9) | 50 (1) | 20 (7) | 48 (2) | 22 (7) |
| *Occupational status* |  |  |  |  |  |  |  |  |  |  |  |  |  |  |
| Regular employee | 2003 (63) | 115 (56) | 2757 (64) | 262 (66) | 2371 (59) | 228 (58) | 1868 (48) | 226 (40) | 1773 (50) | 123 (36) | 1996 (47) | 121 (40) | 1549 (49) | 103 (34) |
| Part-time or temporary worker | 69 (2) | 2 (1) | 92 (2) | 6 (2) | 304 (8) | 33 (8) | 475 (12) | 55 (10) | 560 (16) | 57 (17) | 631 (15) | 35 (12) | 458 (14) | 31 (10) |
| Business owner or family business | 231 (7) | 18 (9) | 148 (3) | 16 (4) | 241 (6) | 34 (9) | 239 (6) | 41 (7) | 231 (7) | 26 (8) | 227 (5) | 18 (6) | 145 (5) | 9 (3) |
| Unemployed | 85 (3) | 8 (4) | 99 (2) | 14 (4) | 130 (3) | 15 (4) | 284 (7) | 36 (6) | 239 (7) | 13 (4) | 376 (9) | 25 (8) | 250 (8) | 18 (6) |
| Student | 740 (23) | 49 (24) | 1153 (27) | 69 (17) | 805 (20) | 42 (11) | 853 (22) | 74 (13) | 634 (18) | 48 (14) | 795 (19) | 36 (12) | 632 (20) | 66 (22) |
| Missing | 47 (1) | 12 (6) | 53 (1) | 30 (8) | 163 (4) | 43 (11) | 203 (5) | 138 (24) | 83 (2) | 74 (22) | 187 (4) | 64 (21) | 139 (4) | 73 (24) |
| *Region of residence* |  |  |  |  |  |  |  |  |  |  |  |  |  |  |
| Hokkaido | 69 (2) | 10 (5) | 145 (3) | 12 (3) | 144 (4) | 15 (4) | 135 (3) | 18 (3) | 119 (3) | 16 (5) | 128 (3) | 5 (2) | 98 (3) | 14 (5) |
| Tohoku | 258 (8) | 15 (7) | 461 (11) | 34 (9) | 201 (5) | 21 (5) | 302 (8) | 43 (8) | 241 (7) | 28 (8) | 323 (8) | 20 (7) | 191 (6) | 18 (6) |
| Kanto | 1149 (36) | 70 (34) | 1518 (35) | 134 (34) | 1406 (35) | 142 (36) | 1384 (35) | 196 (34) | 1347 (38) | 79 (23) | 1403 (33) | 116 (39) | 1197 (38) | 84 (28) |
| Chubu | 570 (18) | 38 (19) | 908 (21) | 92 (23) | 944 (24) | 88 (22) | 786 (20) | 101 (18) | 671 (19) | 81 (24) | 806 (19) | 59 (20) | 589 (19) | 69 (23) |
| Kinki | 514 (16) | 36 (18) | 477 (11) | 64 (16) | 467 (12) | 53 (13) | 585 (15) | 94 (16) | 515 (15) | 54 (16) | 644 (15) | 42 (14) | 507 (16) | 46 (15) |
| Chugoku/Shikoku | 270 (9) | 10 (5) | 360 (8) | 31 (8) | 416 (10) | 39 (10) | 373 (10) | 49 (9) | 237 (7) | 23 (7) | 380 (9) | 23 (8) | 302 (10) | 20 (7) |
| Kyushu/Okinawa | 345 (11) | 25 (12) | 433 (10) | 30 (8) | 436 (11) | 37 (9) | 357 (9) | 69 (12) | 390 (11) | 60 (18) | 528 (13) | 34 (11) | 289 (9) | 49 (16) |
| *Area of Residence: Population Size and Density* |  |  |  |  |  |  |  |  |  |  |  |  |  |  |
| Non-densely inhabited area | 1133 (36) | 67 (33) | 1404 (33) | 165 (42) | 1177 (29) | 128 (32) | 1322 (34) | 200 (35) | 1274 (36) | 161 (47) | 1410 (33) | 105 (35) | 934 (29) | 121 (40) |
| < 200,000 | 734 (23) | 48 (24) | 1290 (30) | 104 (26) | 1021 (25) | 102 (26) | 878 (22) | 128 (22) | 749 (21) | 55 (16) | 926 (22) | 70 (23) | 739 (23) | 54 (18) |
| 200,000 to < 1,000,000 | 603 (19) | 39 (19) | 825 (19) | 72 (18) | 1034 (26) | 91 (23) | 844 (22) | 129 (23) | 817 (23) | 66 (19) | 1058 (25) | 64 (21) | 890 (28) | 81 (27) |
| ≥ 1,000,000 | 705 (22) | 50 (25) | 783 (18) | 56 (14) | 782 (19) | 74 (19) | 878 (22) | 113 (20) | 680 (19) | 59 (17) | 818 (19) | 60 (20) | 610 (19) | 44 (15) |

When accounting also for married men, the proportion excluded due to missing data on relationship status was 3% (204/7806) in 1987, 5% (397/8534) in 1992, 5% (395/7305) in 1997, 8% (570/7524) in 2002, 5% (341/6530) in 2005, 4% (299/7468) in 2010 and 5% (300/5632) in 2015.

**STable 3** Number of men and women who were included in the analyses from each round of the National Fertility Survey 1987-2015.

|  | *Women* | *Men* | *Total* |
| --- | --- | --- | --- |
| *Survey Year* | *n (n unmarried)* | *n (n unmarried)* | *n (n unmarried)* |
| 1987 | 8490 (2560) | 7806 (3175) | 16296 (5735) |
| 1992 | 9141 (3706) | 8534 (4302) | 17675 (8008) |
| 1997 | 8000 (3722) | 7305 (4014) | 15305 (7736) |
| 2002 | 8130 (3601) | 7524 (3922) | 15654 (7523) |
| 2005 | 7328 (3429) | 6530 (3520) | 13858 (6949) |
| 2010 | 8091 (3983) | 7468 (4212) | 15559 (8195) |
| 2015 | 6051 (3042) | 5632 (3173) | 11683 (6215) |

The married participants were recruited as couples for participation in the sub-survey for married couples. As such, the actual number of questionnaires used in the survey is lower than that reported here as the wives provided information also about their husbands.

**STable 4.** Definitions and categorization of socioeconomic and regional variables used for describing population characteristics of participants in the National Fertility Survey 2015.

| *Variable* | *Categorization* | *Missing, weighted %* |
| --- | --- | --- |
| Education | High school or less; vocational school or short-term college; undergraduate studies; graduate studies. | 1.0 |
| Occupational status | Regular employee; part-time or temporary worker; business owner or member of family business; unemployed; student. | 3.6 |
| Working hours (per week)^a^ | 0-40; 41-59; ≥60 | 2.2 |
| Annual income in 10,000 Japanese Yen (JPY)^b^ | 0-99; 100-299; 300-499; 500-799; ≥800 | 3.0 |
| Wish to get married in lifetime | Yes; No | 0.7 |
| Heterosexual experience^c^ | Yes; No | 1.5 |
| Region of residence^d^ | Hokkaido; Tohoku; Kanto; Chubu; Kinki; Chugoku/Shikoku; Kyushu/Okinawa | 0 |
| Population density and size of residence | Non-densely inhabited district; district with less than 200,000 inhabitants; between 200,000 and 1,000,000 inhabitants; more than 1,000,000 inhabitants^14^ | 0 |

a. Only assessed for those with regular employment.

b. 10,000 JPY was approximately 82 Euro as of July 2019. Income was categorized according to the individual’s revenue.

c. We assumed that all married participants and unmarried participants who had been previously married had heterosexual experience. The sub-survey of unmarried participants included the question, “Have you ever had sexual intercourse with someone of the opposite sex?” Never-married participants who answered “no” to this question were categorized as sexually inexperienced and those who answered “yes” were categorized as having heterosexual experience.

d. The seven regions constitute geographically clustered prefectures (the highest administrative divisions of Japan) and are often used in discussion of regional economic and policy issues in the country.

**STable 5.** Heterosexual relationship status among women, standardized to the age-distribution in 2015, 1987-2015. Numbers are shown in percent (95% CI). Raw data for Figures 1 and 2.

| *Age Group* | *Relationship Status* | *1987* | *1992* | *1997* | *2002* | *2005* | *2010* | *2015* | *aOR (95% CI)* |
| --- | --- | --- | --- | --- | --- | --- | --- | --- | --- |
| 18-24 | Married | 12.3 (11.1-13.6) | 10.0 (9.0-11.1) | 8.7 (7.7-9.8) | 7.9 (6.9-9.0) | 7.5 (6.5-8.7) | 6.9 (5.9-8.0) | 5.9 (4.8-7.1) | 0.971 (0.965-0.977) |
|  | In a relationship | 27.7 (25.8-29.6) | 34.6 (32.9-36.4) | 33.5 (31.7-35.4) | 36.8 (34.7-39.0) | 35.1 (33.0-37.3) | 33.4 (31.4-35.5) | 28.6 (26.3-30.9) | 1.002 (0.999-1.006) |
|  | Single | 60.0 (58.0-62.0) | 55.4 (53.6-57.2) | 57.7 (55.8-59.7) | 55.3 (53.1-57.4) | 57.4 (55.1-59.6) | 59.7 (57.5-61.8) | 65.6 (63.1-67.9) | 1.007 (1.003-1.011) |
| 25-29 | Married | 64.3 (61.8-66.7) | 55.6 (53.3-57.8) | 47.8 (45.6-50.1) | 41.1 (39.0-43.3) | 37.7 (35.4-40.0) | 36.7 (34.4-39.1) | 35.9 (33.3-38.6) | 0.954 (0.950-0.959) |
|  | In a relationship | 12.7 (11.1-14.6) | 17.6 (15.8-19.5) | 21.6 (19.7-23.6) | 27.6 (25.5-29.8) | 28.0 (25.7-30.4) | 24.5 (22.4-26.8) | 22.2 (19.9-24.7) | 1.026 (1.021-1.031) |
|  | Single | 23.0 (20.8-25.3) | 26.9 (24.8-29.0) | 30.6 (28.5-32.8) | 31.3 (29.1-33.5) | 34.3 (31.9-36.8) | 38.8 (36.3-41.3) | 41.9 (39.2-44.7) | 1.031 (1.026-1.036) |
| 30-34 | Married | 84.9 (82.8-86.8) | 80.5 (78.4-82.4) | 73.7 (71.4-75.8) | 66.5 (64.3-68.6) | 62.7 (60.4-64.9) | 60.4 (58.2-62.6) | 60.6 (58.0-63.2) | 0.951 (0.947-0.956) |
|  | In a relationship | 3.8 (2.9-5.0) | 4.7 (3.7-5.9) | 7.6 (6.4-9.1) | 11.6 (10.2-13.3) | 11.8 (10.3-13.5) | 11.3 (9.9-12.9) | 9.1 (7.6-10.9) | 1.039 (1.031-1.046) |
|  | Single | 11.3 (9.6-13.2) | 14.8 (13.1-16.8) | 18.7 (16.8-20.7) | 21.9 (20.0-23.9) | 25.6 (23.5-27.7) | 28.2 (26.2-30.3) | 30.2 (27.8-32.8) | 1.045 (1.039-1.051) |
| 35-39 | Married | - | 86.5 (84.7-88.2) | 83.0 (80.9-84.9) | 77.4 (75.2-79.6) | 73.8 (71.4-76.0) | 69.8 (67.8-71.7) | 69.6 (67.3-71.8) | 0.953 (0.947-0.959) |
|  | In a relationship | - | 2.3 (1.6-3.2) | 3.9 (3.0-5.1) | 7.0 (5.7-8.5) | 6.6 (5.4-8.1) | 6.5 (5.5-7.8) | 6.0 (4.9-7.4) | 1.038 (1.026-1.049) |
|  | Single | - | 11.2 (9.7-12.9) | 13.2 (11.4-15.1) | 15.5 (13.7-17.6) | 19.6 (17.5-21.8) | 23.7 (21.8-25.6) | 24.4 (22.3-26.6) | 1.046 (1.039-1.054) |
| 18-39 | Married | - | 57.7 (56.7-58.7) | 53.1 (52.0-54.2) | 48.2 (47.1-49.3) | 45.5 (44.4-46.6) | 43.4 (42.4-44.5) | 42.9 (41.8-44.1) | 0.960 (0.957-0.963) |
|  | In a relationship | - | 14.9 (14.2-15.7) | 16.6 (15.8-17.4) | 20.6 (19.7-21.5) | 20.1 (19.2-21.1) | 18.8 (17.9-19.7) | 16.3 (15.4-17.3) | 1.009 (1.005-1.012) |
|  | Single | - | 27.4 (26.5-28.4) | 30.3 (29.3-31.4) | 31.2 (30.2-32.3) | 34.4 (33.3-35.6) | 37.8 (36.7-38.9) | 40.7 (39.5-42.0) | 1.030 (1.027-1.033) |

aORs were calculated with logistic regression, accounting for sample weights. Analyses were performed separately for each relationship status category, using the investigated category as the dependent variable (1=yes, 0=no), and survey year and age as continuous independent variables. aORs represent the change in the relationship status category per year during the study period.

aOR, age-adjusted odds ratio.

**STable 6.** Heterosexual relationship status among men, standardized to the age-distribution in 2015, 1987-2015. Prevalence is shown in percent (95% CI). Raw data for Figures 1 and 3.

| *Age Group* | *Relationship Status* | *1987* | *1992* | *1997* | *2002* | *2005* | *2010* | *2015* | *aOR (95% CI)* |
| --- | --- | --- | --- | --- | --- | --- | --- | --- | --- |
| 18-24 | Married | 5.3 (4.5-6.3) | 4.8 (4.1-5.6) | 4.8 (4.1-5.6) | 4.8 (4.0-5.7) | 4.5 (3.6-5.7) | 4.1 (3.4-5.0) | 3.6 (2.7-4.7) | 0.988 (0.979-0.997) |
|  | In a relationship | 22.9 (21.2-24.8) | 26.0 (24.4-27.7) | 26.3 (24.5-28.2) | 26.2 (24.4-28.2) | 26.1 (23.9-28.4) | 22.2 (20.3-24.2) | 20.5 (18.4-22.8) | 0.994 (0.990-0.999) |
|  | Single | 71.8 (69.8-73.6) | 69.2 (67.4-70.9) | 68.9 (67.0-70.7) | 69.0 (67.0-70.9) | 69.4 (67.0-71.7) | 73.7 (71.6-75.7) | 75.9 (73.5-78.1) | 1.008 (1.004-1.012) |
| 25-29 | Married | 37.6 (35.3-40.0) | 34.3 (32.2-36.5) | 31.7 (29.6-33.8) | 28.7 (26.9-30.7) | 27.2 (25.2-29.4) | 27.2 (25.1-29.4) | 26.2 (23.9-28.7) | 0.979 (0.975-0.984) |
|  | In a relationship | 16.6 (14.7-18.6) | 23.2 (21.2-25.3) | 21.8 (19.8-23.9) | 24.7 (22.6-27.0) | 24.9 (22.7-27.2) | 22.8 (20.8-25.0) | 18.7 (16.5-21.2) | 1.006 (1.001-1.011) |
|  | Single | 45.8 (43.3-48.4) | 42.5 (40.1-44.9) | 46.5 (44.2-48.9) | 46.5 (44.1-49.0) | 47.9 (45.4-50.5) | 50.0 (47.5-52.5) | 55.1 (52.2-57.9) | 1.013 (1.009-1.018) |
| 30-34 | Married | 68.3 (65.9-70.7) | 63.9 (61.5-66.2) | 58.8 (56.2-61.3) | 53.4 (51.0-55.7) | 50.6 (48.2-52.9) | 50.2 (47.9-52.5) | 50.6 (47.9-53.2) | 0.970 (0.966-0.975) |
|  | In a relationship | 4.7 (3.7-6.1) | 7.8 (6.5-9.4) | 10.2 (8.7-12.1) | 10.7 (9.2-12.4) | 11.5 (9.9-13.3) | 12.1 (10.6-13.8) | 10.1 (8.5-11.9) | 1.026 (1.019-1.034) |
|  | Single | 26.9 (24.7-29.3) | 28.3 (26.1-30.6) | 31.0 (28.6-33.5) | 35.9 (33.6-38.3) | 37.9 (35.5-40.4) | 37.7 (35.4-40.0) | 39.3 (36.7-42.0) | 1.023 (1.018-1.028) |
| 35-39 | Married | - | 77.0 (74.8-79.0) | 73.2 (70.7-75.5) | 68.5 (65.9-71.0) | 65.2 (62.8-67.6) | 61.0 (58.7-63.1) | 61.6 (59.1-64.0) | 0.965 (0.960-0.971) |
|  | In a relationship | - | 2.6 (1.9-3.6) | 4.4 (3.3-5.8) | 5.2 (4.0-6.7) | 5.7 (4.6-7.1) | 5.9 (4.9-7.2) | 6.0 (4.8-7.4) | 1.032 (1.019-1.045) |
|  | Single | - | 20.4 (18.4-22.5) | 22.4 (20.2-24.8) | 26.3 (23.9-28.9) | 29.1 (26.8-31.5) | 33.1 (31.0-35.3) | 32.4 (30.1-34.9) | 1.032 (1.026-1.038) |
| 18-39 | Married | - | 45.0 (44.0-46.0) | 42.2 (41.1-43.3) | 39.0 (37.9-40.0) | 37.0 (35.9-38.1) | 35.6 (34.7-36.7) | 35.5 (34.4-36.7) | 0.974 (0.971-0.977) |
|  | In a relationship | - | 14.6 (13.9-15.4) | 15.5 (14.6-16.4) | 16.4 (15.5-17.3) | 16.7 (15.8-17.7) | 15.4 (14.6-16.3) | 13.6 (12.7-14.6) | 0.999 (0.995-1.002) |
|  | Single | - | 40.4 (39.3-41.4) | 42.3 (41.2-43.5) | 44.6 (43.5-45.8) | 46.2 (45.0-47.5) | 48.9 (47.8-50.1) | 50.8 (49.5-52.1) | 1.022 (1.019-1.025) |

aORs were calculated with logistic regression, accounting for sample weights. Analyses were performed separately for each relationship status category, using the investigated category as the dependent variable (1=yes, 0=no), and survey year and age (in years) as continuous independent variables. aORs represent the change in the relationship status category per year during the study period.

aOR, age-adjusted odds ratio.

**STable 7.** Proportion of men and women who are married, in a heterosexual relationship, single with interest in romantic relationships and single with no interest in romantic relationships, respectively (2010 and 2015). Numbers are shown in percent. Raw data for Figure 4 and Figure S1.

|  | *2010* | | | | *2015* | | | |
| --- | --- | --- | --- | --- | --- | --- | --- | --- |
| *Women* | *Married* | *In a  relationship* | *Single  & interest* | *Single  & no interest* | *Married* | *In a  relationship* | *Single  & interest* | *Single  & no interest* |
| 18-24 y | 6.9 | 33.4 | 28.4 | 31.3 | 5.9 | 28.6 | 28.2 | 37.4 |
| 25-29 y | 36.7 | 24.5 | 22.4 | 16.4 | 35.9 | 22.2 | 23.0 | 18.9 |
| 30-34 y | 60.4 | 11.3 | 15.7 | 12.5 | 60.6 | 9.1 | 15.9 | 14.4 |
| 35-39 y | 69.8 | 6.5 | 11.1 | 12.6 | 69.6 | 6.0 | 10.9 | 13.5 |
| 18-39 y | 43.4 | 18.8 | 19.2 | 18.6 | 42.9 | 16.3 | 19.4 | 21.4 |
| *Men* |  |  |  |  |  |  |  |  |
| 18-24 y | 4.1 | 22.2 | 38.2 | 35.5 | 3.6 | 20.5 | 39.3 | 36.6 |
| 25-29 y | 27.2 | 22.8 | 28.1 | 21.9 | 26.2 | 18.7 | 28.2 | 26.8 |
| 30-34 y | 50.2 | 12.1 | 21.4 | 16.2 | 50.6 | 10.1 | 19.9 | 19.5 |
| 35-39 y | 61.0 | 5.9 | 17.8 | 15.3 | 61.6 | 6.0 | 15.4 | 17.1 |
| 18-39 y | 35.6 | 15.4 | 26.4 | 22.5 | 35.5 | 13.6 | 25.7 | 25.1 |

Estimates for 2010 are standardized to the age-distribution of 2015.

**STable 8.** Population characteristics of women by heterosexual relationship status and interest in romantic relationships in 2015, standardized to the age-distribution of married women. Numbers are shown in percent and the relative risks are shown in the brackets (reference group is married individuals unless otherwise indicated).

|  | *Married* | *In a relationship* | *Single & interest* | *Single & no interest* | *p-value* |
| --- | --- | --- | --- | --- | --- |
| *Age group* |  |  |  |  | 1.0000 |
| 18-24 | 3.7 | 3.7 | 3.7 | 3.7 |  |
| 25-29 | 17.3 | 17.3 | 17.3 | 17.3 |  |
| 30-34 | 33.9 | 33.9 | 33.9 | 33.9 |  |
| 35-39 | 45.1 | 45.1 | 45.1 | 45.1 |  |
| *Education* |  |  |  |  | 0.0017 |
| High school of less | 34.3 (ref) | 35.4 (1.03) | 25.2 (0.73) | 40.3 (1.17) |  |
| Vocational school or short college | 39.3 (ref) | 39.9 (1.02) | 41.3 (1.05) | 34.7 (0.88) |  |
| Undergraduate studies | 24.2 (ref) | 23.8 (0.98) | 32.2 (1.33) | 22.4 (0.93) |  |
| Graduate studies | 2.2 (ref) | 0.8 (0.36) | 1.3 (0.59) | 2.6 (1.18) |  |
| *Occupational status* |  |  |  |  | < 0.0001 |
| Regular employee | 25.9 (ref) | 55.6 (2.15) | 59.8 (2.31) | 42 (1.62) |  |
| Part-time or temporary worker | 32 (ref) | 31.3 (0.98) | 27.9 (0.87) | 36.2 (1.13) |  |
| Business owner or family business | 5 (ref) | 5.1 (1.02) | 1.8 (0.36) | 4.2 (0.84) |  |
| Unemployed | 36.9 (ref) | 7.1 (0.19) | 8.9 (0.24) | 15.4 (0.42) |  |
| Student | 0.2 (ref) | 0.9 (4.5) | 1.6 (8) | 2.2 (11) |  |
| *Working hours (per week)^a^* |  |  |  |  | 0.0001 |
| 0-40 | 55.3 (ref) | 31.4 (0.57) | 39.2 (0.71) | 44.9 (0.81) |  |
| 41-60 | 43.7 (ref) | 65.5 (1.5) | 56 (1.28) | 51.4 (1.18) |  |
| > 60 | 1 (ref) | 3.1 (3.1) | 4.8 (4.8) | 3.6 (3.6) |  |
| *Annual income (in JPY 10,000s)* |  |  |  |  | < 0.0001 |
| 0 | 61.8 (ref) | 19.5 (0.32) | 19.7 (0.32) | 31.6 (0.51) |  |
| 100-299 | 23.3 (ref) | 44.9 (1.93) | 49.2 (2.11) | 47.5 (2.04) |  |
| 300-499 | 11.9 (ref) | 30.1 (2.53) | 26.4 (2.22) | 17.9 (1.5) |  |
| 500-799 | 2.6 (ref) | 5.2 (2) | 4.7 (1.81) | 3 (1.15) |  |
| ≥ 800 | 0.4 (ref) | 0.3 (0.75) | 0 (0) | 0 (0) |  |
| *Wish to get married in lifetime* |  |  |  |  | < 0.0001 |
| No | - | 8.5 (ref) | 2.2 (0.26) | 46 (5.41) |  |
| Yes | - | 91.5 (ref) | 97.8 (1.07) | 54 (0.59) |  |
| *Heterosexual experience* |  |  |  |  | < 0.0001 |
| Experienced | 100 (ref) | 95.2 (0.95) | 69.1 (0.69) | 56.1 (0.56) |  |
| No experience | 0 | 4.8 (ref) | 30.9 (6.44) | 43.9 (9.15) |  |
| *Region of Residence* |  |  |  |  | 0.1365 |
| Hokkaido | 3.4 (ref) | 4.1 (1.21) | 5 (1.47) | 4.7 (1.38) |  |
| Tohoku | 6.7 (ref) | 11 (1.64) | 5.9 (0.88) | 9.5 (1.42) |  |
| Kanto | 31.6 (ref) | 28.3 (0.9) | 33.4 (1.06) | 28.7 (0.91) |  |
| Chubu | 21 (ref) | 19.8 (0.94) | 18.5 (0.88) | 16.9 (0.8) |  |
| Kinki | 15.1 (ref) | 18.5 (1.23) | 16 (1.06) | 16.4 (1.09) |  |
| Chugoku/Shikoku | 11 (ref) | 7.8 (0.71) | 8.1 (0.74) | 9.6 (0.87) |  |
| Kyushu/Okinawa | 11.2 (ref) | 10.5 (0.94) | 13.1 (1.17) | 14.2 (1.27) |  |
| *Area of Residence: Population Size and Density* |  |  |  |  | 0.2757 |
| Non-densely inhabited area | 28.5 (ref) | 30.1 (1.06) | 26.2 (0.92) | 31.5 (1.11) |  |
| < 200,000 | 24.5 (ref) | 22.7 (0.93) | 19.6 (0.8) | 25 (1.02) |  |
| 200,000 to < 1,000,000 | 27.7 (ref) | 30.1 (1.09) | 31.9 (1.15) | 27.2 (0.98) |  |
| ≥ 1,000,000 | 19.3 (ref) | 17.1 (0.89) | 22.2 (1.15) | 16.3 (0.84) |  |
| N unweighted/weighted | 3009/1294 | 887/381.3 | 810/348.2 | 885/380.5 |  |

a. Among those with regular employment. S**Table 9.** Population characteristics of men by heterosexual relationship status and interest in relationships in 2015, standardized to the age-distribution of married men. Numbers are shown in percent and the relative risks are shown in the brackets (reference group is married individuals unless otherwise indicated).

|  | Married | In a relationship | Single & interest | Single & no interest | *p*-value |
| --- | --- | --- | --- | --- | --- |
| *Age group* |  |  |  |  | 1.0000 |
| 18-24 | 2.8 | 2.8 | 2.8 | 2.8 |  |
| 25-29 | 15.2 | 15.2 | 15.2 | 15.2 |  |
| 30-34 | 33.9 | 33.9 | 33.9 | 33.9 |  |
| 35-39 | 48.1 | 48.1 | 48.1 | 48.1 |  |
| *Education* |  |  |  |  | 0.0052 |
| High school of less | 41.5 (ref) | 36.8 (0.89) | 38.5 (0.93) | 46.5 (1.12) |  |
| Vocational school or short college | 18.8 (ref) | 17.4 (0.93) | 18.8 (1) | 22.7 (1.21) |  |
| Undergraduate studies | 33.7 (ref) | 38 (1.13) | 34.5 (1.02) | 26.8 (0.8) |  |
| Graduate studies | 6 (ref) | 7.8 (1.3) | 8.2 (1.37) | 3.9 (0.65) |  |
| *Occupational Status* |  |  |  |  | < 0.0001 |
| Regular employee | 85.8 (ref) | 69.5 (0.81) | 62.2 (0.72) | 51.2 (0.6) |  |
| Part-time or temporary worker | 4.1 (ref) | 12.7 (3.1) | 18.4 (4.49) | 22.8 (5.56) |  |
| Business owner or family business | 9.2 (ref) | 10 (1.09) | 9.4 (1.02) | 7.4 (0.8) |  |
| Unemployed | 0.7 (ref) | 6.1 (8.71) | 8 (11.43) | 17.2 (24.57) |  |
| Student | 0.2 (ref) | 1.7 (8.5) | 2 (10) | 1.4 (7) |  |
| *Working Hours (per week)^a^* |  |  |  |  | 0.1091 |
| 0-40 | 21.4 (ref) | 19.2 (0.9) | 22.2 (1.04) | 25.1 (1.17) |  |
| 41-60 | 64 (ref) | 70.6 (1.1) | 66.4 (1.04) | 67.8 (1.06) |  |
| > 60 | 14.5 (ref) | 10.2 (0.7) | 11.4 (0.79) | 7 (0.48) |  |
| *Annual Income (in JPY 10,000s)* |  |  |  |  | < 0.0001 |
| 0 | 6.8 (ref) | 17 (2.5) | 19 (2.79) | 29.6 (4.35) |  |
| 100-299 | 16.6 (ref) | 22.5 (1.36) | 28.3 (1.7) | 33.8 (2.04) |  |
| 300-499 | 44.5 (ref) | 41.3 (0.93) | 37.3 (0.84) | 29.1 (0.65) |  |
| 500-799 | 27.2 (ref) | 17.3 (0.64) | 14.5 (0.53) | 7.3 (0.27) |  |
| ≥ 800 | 5 (ref) | 2 (0.4) | 0.9 (0.18) | 0.2 (0.04) |  |
| *Wish to Get Married in Lifetime* |  |  |  |  | < 0.0001 |
| No | - | 1.8 (ref) | 3.1 (1.72) | 43.9 (24.39) |  |
| Yes | - | 98.2 (ref) | 96.9 (0.99) | 56.1 (0.57) |  |
| *Heterosexual Experience* |  |  |  |  | < 0.0001 |
| Experienced | 100 (ref) | 96.5 (0.97) | 74.9 (0.75) | 61.4 (0.61) |  |
| No experience | 0 | 3.5 (ref) | 25.1 (7.17) | 38.6 (11.03) |  |
| *Region of Residence* |  |  |  |  | 0.0059 |
| Hokkaido | 3.9 (ref) | 3.3 (0.85) | 2.2 (0.56) | 4.3 (1.1) |  |
| Tohoku | 6.6 (ref) | 6.6 (1) | 6 (0.91) | 8.5 (1.29) |  |
| Kanto | 30.7 (ref) | 33.6 (1.09) | 37.4 (1.22) | 38.3 (1.25) |  |
| Chubu | 20.5 (ref) | 15.3 (0.75) | 22.1 (1.08) | 15.9 (0.78) |  |
| Kinki | 14.8 (ref) | 21.4 (1.45) | 14.1 (0.95) | 16.4 (1.11) |  |
| Chugoku/Shikoku | 11.5 (ref) | 10.6 (0.92) | 9.6 (0.83) | 8.7 (0.76) |  |
| Kyushu/Okinawa | 11.9 (ref) | 9.3 (0.78) | 8.6 (0.72) | 7.9 (0.66) |  |
| *Area of Residence: Population Size* and Density |  |  |  |  | 0.8177 |
| Non-densely inhabited area | 28.8 (ref) | 28.9 (1) | 31.3 (1.09) | 28.1 (0.98) |  |
| < 200,000 | 25.1 (ref) | 24.8 (0.99) | 22.3 (0.89) | 23.9 (0.95) |  |
| 200,000 to < 1,000,000 | 27.7 (ref) | 25.1 (0.91) | 26.3 (0.95) | 29.8 (1.08) |  |
| ≥ 1,000,000 | 18.4 (ref) | 21.2 (1.15) | 20 (1.09) | 18.3 (0.99) |  |
| *N* unweighted/weighted | 2459/873.2 | 671/238.3 | 1046/371.4 | 1023/363.3 |  |

a. Among those with regular employment.

**STable 10.** Proportion of the population that is single in Natsal-3 (Britain, 2010-2012), the General Social Survey (US, 2012-2018) and the National Fertility Survey (Japan, 2015). Numbers are shown in percent (95% CI).

|  | *Women* | | | *Men* | | |
| --- | --- | --- | --- | --- | --- | --- |
|  | *Natsal-3 (Britain)* | *General Social Survey (US)* | *National Fertility Survey (Japan)* | *Natsal-3 (Britain)* | *General Social Survey (US)* | *National Fertility Survey (Japan)* |
| 18-24 years | 41.5 (38.7 - 44.3) | 62.6 (55.3 - 69.4) | 65.6 (63.1-67.9) | 52.6 (49.4 - 55.8) | 81.4 (75.4 - 86.2) | 75.9 (73.5-78.1) |
| 25-29 years | 23.6 (21.1 - 26.2) | 25.2 (19.8 - 31.4) | 41.9 (39.2-44.7) | 32.5 (29.0 - 36.2) | 55.8 (47.6 - 63.6) | 55.1 (52.2-57.9) |
| 30-34 years | 16.3 (14.2 - 18.7) | 20.0 (15.8 - 24.9) | 30.2 (27.8-32.8) | 14.7 (12.1 - 17.8) | 35.9 (28.1 - 44.6) | 39.3 (36.7-42.0) |
| 35-39 years | 14.0 (11.3 - 17.2) | 16.6 (12.5 - 21.7) | 24.4 (22.3-26.6) | 11.8 (9.1 - 15.2) | 22.0 (15.9 - 29.7) | 32.4 (30.1-34.9) |
| 18-39 years | 25.5 (24.0 – 27.0) | 31.8 (28.5 - 35.2) | 40.7 (39.5-42.0) | 30.5 (28.7 - 32.3) | 54.0 (49.7 - 58.3) | 50.8 (49.5-52.1) |

**SFigure 1.** Heterosexual relationship status and interest in romantic relationships among women and men in 2010. The estimates are standardized to the age-distribution of 2015.


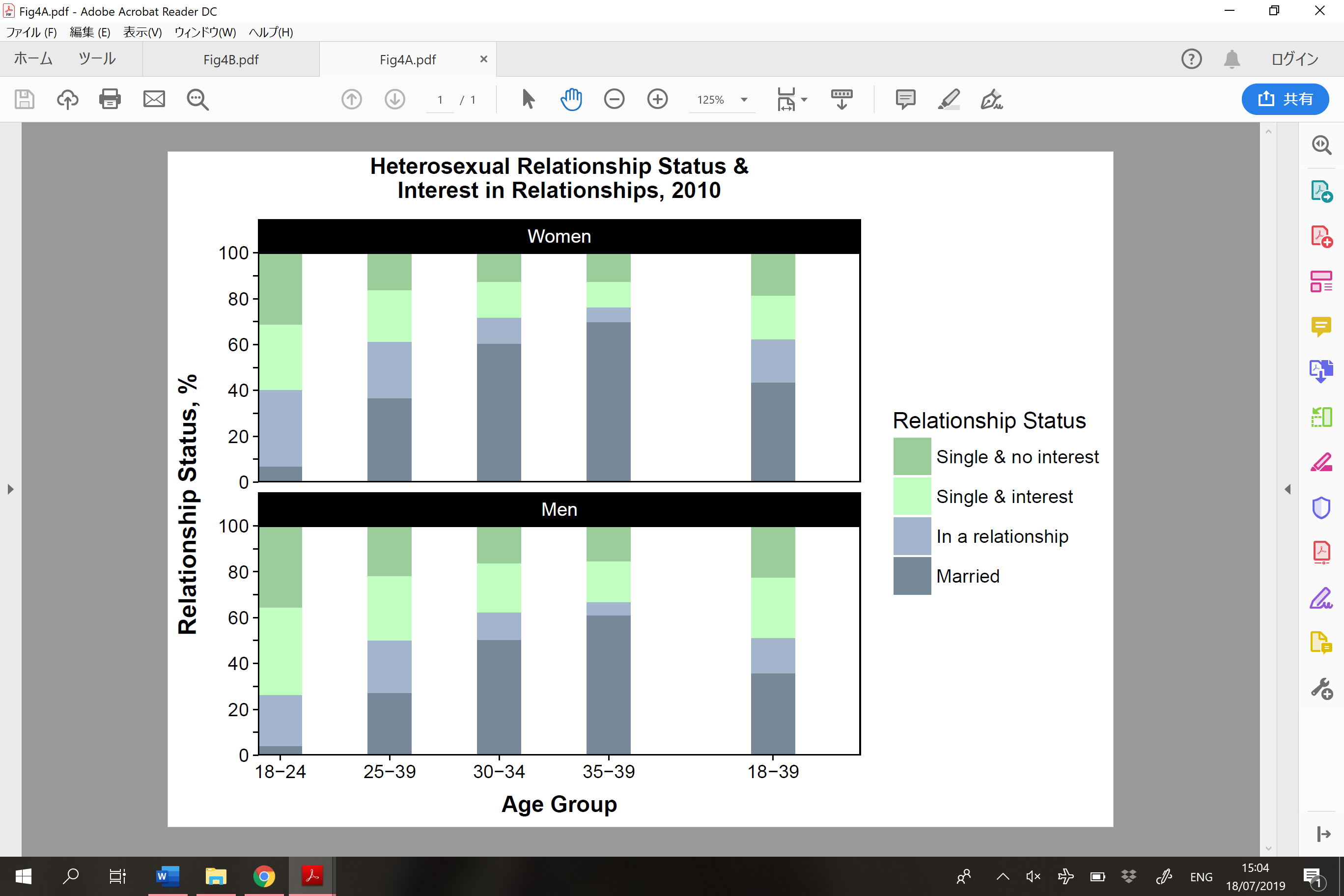


**SFigure 2.** Reasons for staying single among single women and men with no interest in romantic relationships, 2015. Bars represent the weighted proportion.

|  | **Reason for staying single** | **Women,%** | **Men,%** |
| --- | --- | --- | --- |
| A | Still too young for marriage | 19.3 | 17.2 |
| B | Do not feel the need to marry yet | 37.1 | 33.7 |
| C | Currently, prefer to focus on work/school | 25.2 | 21.1 |
| D | Currently, want to spend time on hobbies and pursuing pleasures | 17.3 | 17.4 |
| E | Do not want to lose the freedom and carefree nature of singlehood | 20.3 | 21.9 |
| F | Still have not met a suitable person | 38.6 | 32.5 |
| G | Not good at interacting/going on dates with persons of the opposite sex | 11.0 | 10.9 |
| H | Do not have enough money for marriage | 4.8 | 14.7 |
| I | Do not have prospects for housing for post-marriage life | 1.2 | 2.0 |
| J | Parents and others around me (probably) do not approve of marriage | 2.7 | 1.2 |
| K | Other | 2.9 | 1.4 |

The answer choice “my marriage is already being planned” is not presented in the figure as there were very few participants who had listed chose this answer.

**References**

1. Ghaznavi C, Sakamoto H, Yoneoka D, Nomura S, Shibuya K, Ueda P: **Trends in heterosexual inexperience among young adults in Japan: analysis of national surveys, 1987-2015**. *BMC Public Health* 2019, **19**(1):355.

2. Mercer CH, Tanton C, Prah P, Erens B, Sonnenberg P, Clifton S, Macdowall W, Lewis R, Field N, Datta J *et al*: **Changes in sexual attitudes and lifestyles in Britain through the life course and over time: findings from the National Surveys of Sexual Attitudes and Lifestyles (Natsal)**. *Lancet* 2013, **382**(9907):1781-1794.

3. **The General Social Survey (GSS)** [<https://gss.norc.org/>]
